# Changing phenotype and disease behaviour of chronic pancreatitis in India: evidence for gene–environment interactions

**DOI:** 10.1017/gheg.2016.13

**Published:** 2016-10-18

**Authors:** P. K. Garg, D. Narayana

**Affiliations:** 1 Department of Gastroenterology, All India Institute of Medical Sciences, New Delhi, India; 2 State Planning Board, Government of Kerala, India

**Keywords:** Chronic pancreatitis, phenotype, SPINK1, tropical pancreatitis

## Abstract

**Background:**

The idiopathic variety of chronic pancreatitis (CP) in India particularly in Kerala state was earlier called ‘tropical pancreatitis’ with peculiar features: early age of onset, severe malnutrition, diabetes and poor prognosis. A change in disease phenotype and behaviour has been observed recently.

**Objective:**

To review the changing profile of CP in India and examine its relationship with environmental influences and socio-economic development.

**Methods:**

Relevant studies on CP in India were reviewed along with social and economic parameters in Kerala over the past 4 decades.

**Results:**

There has been a definite change in the phenotype of CP in India with onset in mid twenties, better nutritional status, and a much better prognosis compared with the reports in 1970s. Genetic susceptibility due to genetic mutations particularly in *SPINK1, CFTR, CTRC*, and *CLDN2/MORC4* genes is the most important factor and not malnutrition or dietary toxins for idiopathic CP suggesting the term ‘tropical pancreatitis’ is a misnomer. We observed a close relationship between socio-economic development and rising income in Kerala with late onset of disease, nutritional status, and better prognosis of CP.

**Conclusion:**

Changing profile of CP in India and better understanding of risk factors provide evidence for gene–environmental interactions in its pathobiology.

One infection-one disease (e.g. tuberculosis, HIV) or one gene-one disease (e.g. cystic fibrosis, haemophilia) hypothesis in disease pathogenesis applies aptly to many conditions but most metabolic diseases such as diabetes and hypertension have complex pathophysiological basis with close interaction between environmental and genetic factors. Chronic pancreatitis (CP) is a disease that occurs due to a variety of causes such as genetic mutation, alcohol, and smoking. Idiopathic CP is diagnosed when no apparent cause is identified. In genetically determined or predisposed diseases, the influence of environmental factors may manifest in different ways: age at onset of disease, phenotype of the disease, natural course of the disease, and response to treatment but such alterations in disease behaviour have not been well studied in most disease conditions. In this review, we examine how the phenotype and disease behaviour of idiopathic CP as reported from India – a disease that is primarily genetically determined – has evolved with changing environmental influences to illustrate complex gene–environment interactions in disease pathobiology.

## What is chronic pancreatitis?

Pancreas is unique in the sense that it comprises two diverse yet intertwined cell types i.e. exocrine and endocrine. Acute pancreatitis is acute inflammation of the pancreas presenting clinically as acute abdominal pain and causing significant local and systemic complications [1]. The most common causes of acute pancreatitis are gallstones and alcohol abuse. If the cause of acute pancreatitis persists, it may lead to recurrent acute pancreatitis. Genetic polymorphism/mutation may also predispose to recurrent acute pancreatitis. Recurrent and chronic inflammation leading to pancreatic injury results in chronic pancreatitis [2].

It can be defined as a pathologic chronic inflammatory disease of the pancreas resulting in progressive parenchymal injury and fibrosis that occurs due to genetic and/or environmental risk factors, and results in variably progressive glandular dysfunction manifesting clinically with abdominal pain and functional impairment in the form of diabetes and steatorrhoea [3]. The diagnosis of CP is made on morphological imaging if there are changes of pancreatic ductal dilatation with or without stricture and/or pancreatic calcification [4]. Calcification represents intraductal calculi either in the main pancreatic duct or side branches. The ductal dilatation and calculi are seen in late stage of the disease. Diagnosis of early CP is difficult as the pancreatic tissue is generally not available for histology. Endoscopic ultrasonography (EUS) is a newer imaging modality that may diagnose early CP with approximately 90% accuracy by demonstrating changes suggestive of parenchymal fibrosis, architectural changes, and ductal abnormalities [5]. The prevalence of CP was reported to be around 10/100 000 in earlier reports [6]. However, more recent studies have shown a higher prevalence of CP in different countries: 13.52/100 000 in China, 26.4/100 000 in France, 45.4/100 000 population in Japan, and 41.8/100 000 in USA [7–9]. The highest prevalence has been reported to be 125/100 000 population from India [10, 11].

## Chronic pancreatitis in India

### Historical perspective

The predominant type of CP that was reported from India was known as tropical calcific pancreatitis (TCP) or simply ‘Tropical Pancreatitis’ (TP). The term, TCP, was used to refer to a special type of CP with large pancreatic calculi that affected very young malnourished individuals who often developed diabetes and had an aggressive course of the disease [12]. Initially described from Indonesia, it has been reported from many other tropical countries [13]. Geevarghese [14] reported a large series of patients with diabetes due to chronic pancreatitis from Kerala, a southern state in India, and described in detail the features peculiar to the type of CP prevalent there. The reported features of TCP included the following: (i) it affected young patients often in their childhood and adolescence, (ii) patients were malnourished with a low body mass index (<18.5), (iii) cassava consumption as a staple diet was causally implicated in its aetiopathogenesis, (iv) most patients were diabetic at presentation, (v) the disease was associated with marked pancreatic ductal dilatation and calcification, and (vi) the natural course was progressively downhill with patients dying in their youth. With these characteristics, tropical calcific pancreatitis was usually considered a special form of aggressive idiopathic CP in India.

### Changing profile of CP in India

**Fig. 1.**
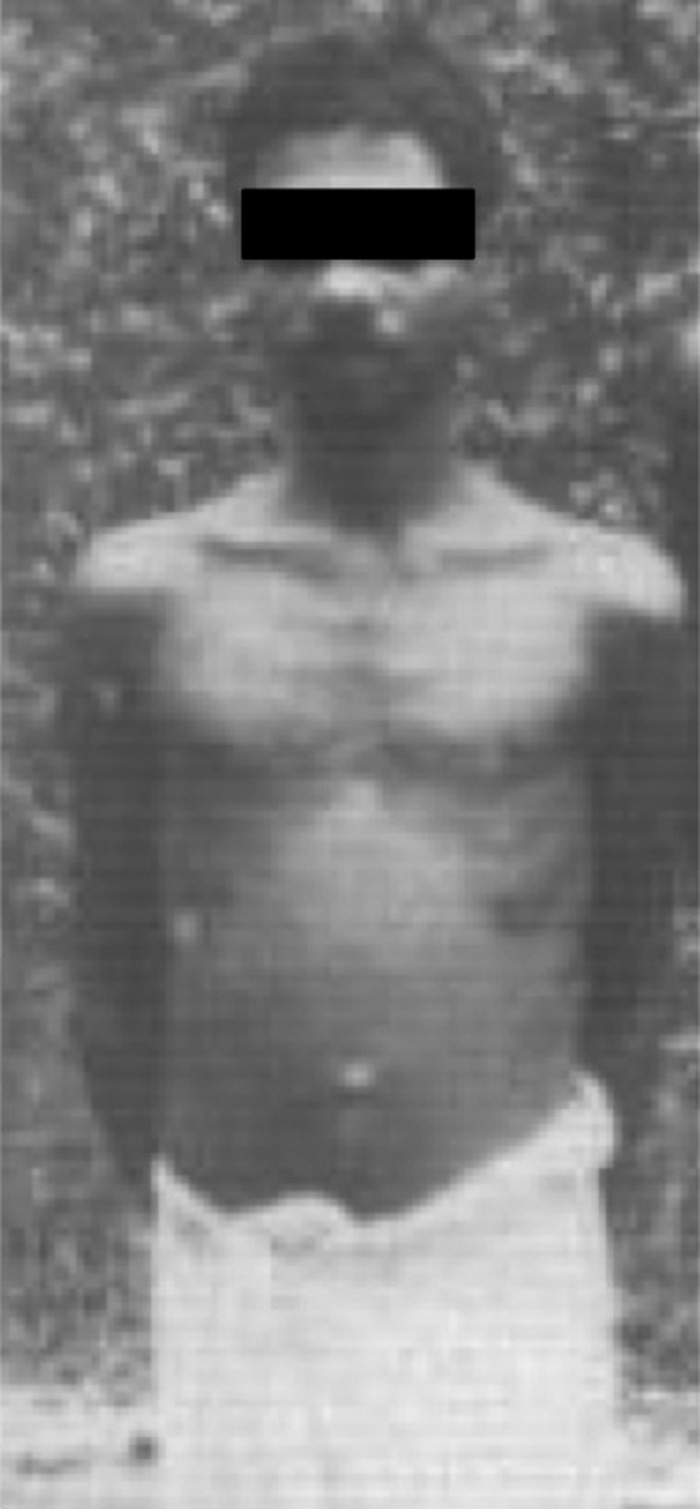
A malnourished young patient from Kerala afflicted with chronic calcific pancreatitis in 1970s (photo courtesy: Dr H Ramesh).

**Fig. 2.**
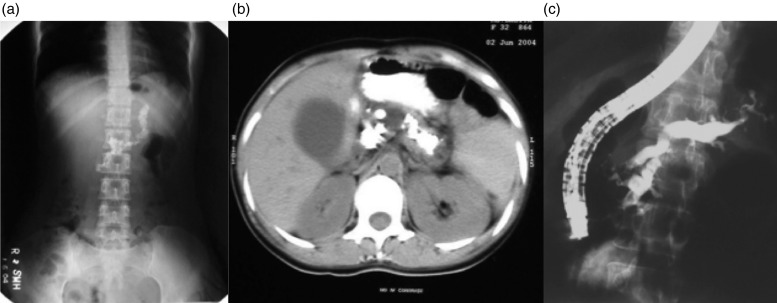
(*a*) Marked pancreatic calcification on a plain x-ray of the abdomen. (*b*) Significant pancreatic atrophy and calculi on a computed tomography scan of the abdomen. (*c*) Dilated pancreatic duct on endoscopic retrograde cholangiopacreatography (ERCP).

**Fig. 3.**
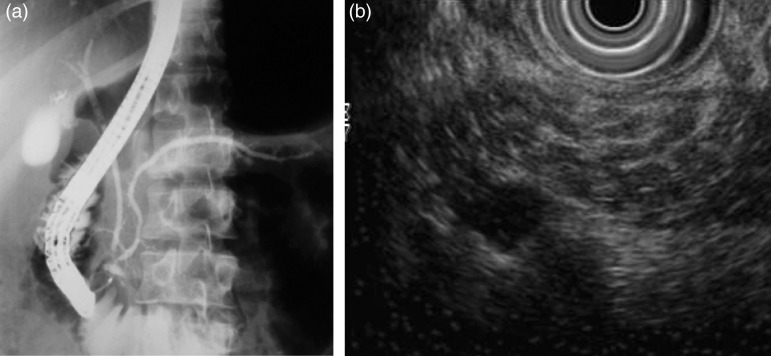
(*a*) Subtle changes of early chronic pancreatitis in the main pancreatic duct on endoscopic retrograde cholangiopancreatography (ERCP). (*b*) Pancreatic parenchymal changes in the form of honeycombing and hyperechoic foci suggestive of early chronic pancreatitis on endoscopic ultrasonography (EUS).

Over the past decade or so, many reports from India have shown that the phenotype of patients with idiopathic CP is quite different from that reported earlier. We will discuss each of the characteristics comparing newer with the older reports:

- Age of onset of CP: The earlier reports showed onset of disease in childhood and adolescents while the mean age of patients with CP has been reported to be in late twenties and early thirties in more recent publications [15, 16]. Nutritional status of patients: it was believed that severe malnutrition particularly protein deficiency could contribute to the development of chronic pancreatitis because most of the patients were malnourished (Fig. 1). Many recent studies have shown that malnutrition is an effect and not a cause of CP. In a case-control study of 120 patients with idiopathic CP, only 20.6% were underweight before the onset of the disease while 67% lost weight after the onset of CP suggesting that malnutrition was an effect and not a cause of CP [17]. Another study in southern Indian patients has shown that malnutrition was not a cause of TCP (idiopathic CP) as only 15% patients were malnourished before the onset of disease and 52% of patients lost weight subsequently [18]. It has been shown that malnutrition results in pancreatic atrophy and insufficiency and not CP thus disproving the nutritional hypothesis [19]. The concept of malnutrition as a likely etiological factor for CP has been discarded [20].
- Cassava as an etiological cause: Another nutritional hypothesis focused on cassava as the cause of CP. Cassava has been a staple diet in Kerala particularly among poor people. It contains certain cyanogenic glycosides, which were thought to be toxic to the pancreas. However, many clinical and experimental studies have shown that cassava is not a cause of CP because: (i) cassava consumption was not found as a risk factor in case-control studies from Kerala [21], (ii) cassava is not consumed in northern India where CP is quite prevalent [15, 16], and (iii) long-term cassava consumption did not produce diabetes or pancreatitis in a rat model [22].
- Morphological features of Pancreas: The previous reports had shown markedly dilated pancreatic duct and large ductal calculi in patients with CP (Fig. 2*a*–*c*). Most patients with CP had diabetes, a feature of advanced disease with pancreatic atrophy and burnt out disease. It is now apparent that these cases did not represent a separate morphological entity but were more advanced cases of the same pathological process, and with advancing knowledge, better imaging modalities, and increasing access to health care, patients with less advanced forms of CP are being diagnosed. Pancreatic calcification was present in only 46.9% of patients with CP in a study from India [16]. In a study, we found that about 50% of patients with recurrent acute pancreatitis developed chronic pancreatitis during follow up [23]. Such a progression suggests that repeated inflammation and acinar cell injury lead to fibrosis, the so-called necrosis-fibrosis hypothesis, in the development of chronic pancreatitis [24]. Thus, the degree of ductal changes and the presence of calculi depend on the stage of the disease at which a patient presents to the clinician (Fig. 3*a*). EUS is a very useful tool to diagnose early chronic pancreatitis (Fig. 3*b*) [5].
- Diabetes: Development of diabetes and steatorrhoea represent advanced stage of the disease with marked loss of functioning pancreatic parenchyma. Earlier reports showed that diabetes was present in up to 90% of patients with TCP [14]. In fact, such patients were diagnosed as having pancreatogenic diabetes rather than CP. However, diabetes was present only in about 1/3rd of patients in our study [15] and in 40.5% of patients with CP in a recent multi-centre study from India [25]. These data also suggest that early form of CP is being diagnosed and the rate of progression to advanced disease may be slower.
- Prognosis of CP: As mentioned above, patients suffering from TCP in Kerala were reported to have a bad prognosis with many of them dying in their twenties with the adage: ‘pancreatitis in childhood, diabetes in adolescence, and death in prime of life’ [26]. This was obviously due to multiple factors: severe malnutrition, recurrent superadded infections, poor control of diabetes and poverty. The National Sample Survey conducted in 1973–1974 reported that Kerala's morbidity was one of the highest in India i.e. 71 per 1000 persons for acute illness and 83 per 1000 persons for chronic illness in the general population. Infections constituted a large share of morbidity, and poor people reported more illness than the rich [27]. However, our recent data have shown a relatively good prognosis in terms of symptom relief and survival. We have shown that the probability of surviving for 35 years after onset of idiopathic CP was 83% i.e. most patients are likely to survive at least up to the age of 60 years [15].

These comparative figures emerging from several recent studies have depicted a vastly different picture of chronic pancreatitis in India. The idea that this type of CP i.e. tropical pancreatitis was different stemmed from the earlier observations of severe malnutrition, diabetes and marked pancreatic calcification. However, in an important multi-centre study from India, only about 3.8% of patients could satisfy the criteria of the so-called TCP [25]. Furthermore, observational studies from Kerala, the state from where TCP was first described, also showed a changing profile of the disease in 2000s compared with that in 1980s with the patients being older, relatively well nourished, diagnosed at early stage of the disease and fewer having diabetes at initial clinical presentation [28]. These differences in the patient profile between earlier and recent reports have been summarized in Table 1. It is quite clear from the data that the phenotype of CP has changed considerably in India. However, we should also take into account the different sets of investigations in different time periods. In more recent reports, investigative modalities such as CT scan, MRI, ERCP and even EUS have been used to diagnose CP but many of these tests were not available in 1960s and 1970s. A lack of sophisticated investigative facilities such as CT scan, MRI and EUS in earlier times could be the reason for only the advanced cases of CP being diagnosed, an issue akin to ‘spectrum bias’ as applicable to a diagnostic test. Nevertheless, the fact that advanced form of CP was diagnosed in childhood in 1970s but in young adults more recently suggests that the onset of disease has indeed advanced because more sophisticated tests would tend to diagnose disease earlier.

**Table 1.** Change in demographics, aetiology, and disease characteristics in patients with Chronic Pancreatitis in India over time

Parameter	Geevarghese [14]	Balakrishnan [28]	Balakrishnan [28]	Midha [15]	Bhasin [16]
Number of patients		220	244	242	155
Age at onset	Childhood	20.7	30.6	24.7	33^a^
Body mass index	<18.5	15.9	20.4	19.6	20.2
Etiology					
Idiopathic	100%	98%	67%	58.9%	41.3%
Alcohol		2%	33%	38.2%	38.1%
Diabetes	90%	77%	59.7%	35.5%	23.4%

All studies were hospital-based cross-sectional studies.

a Age at diagnosis.

Before discussing the likely reasons for the changing profile and disease behaviour of CP, let us first review the current understanding of the pathogenesis of CP.

## Current understanding of the pathogenesis of idiopathic CP in India: genetic mutations

In 1996, a genetic mutation in the *cationic trypsinogen* (*PRSS1*) gene was discovered in patients with hereditary pancreatitis [29]. This paved the way for unravelling the genetic basis for previously idiopathic CP. Mutations in 2 genes namely *CFTR* and *SPINK1* have been shown to be strongly associated with the development of idiopathic CP from different regions of the world [30, 31]. Many reports have also shown mutations in *SPINK1* and *CFTR* genes in patients with CP from both northern and southern India [32–34]. Association with *SPINK1* gene is particularly striking with up to 40% patients with idiopathic CP having *SPINK1* gene mutations, which is much higher than that reported in western patients with CP [30, 33]. Patients from Kerala and southern India also have been shown to have mutations in the *SPINK1* and CFTR genes [35–37]. More recently, associations with polymorphisms/mutation in many other genes such as *CTRC, CLD2/MORC4* have been reported in Indian patients with CP [38–40]. These genetic association studies are summarized in Table 2. Identification of this association has opened a whole new chapter and brought in fresh thinking in our understanding of the aetiopathogenesis of CP in India. From an esoteric disease considered to be causally associated with malnutrition and cassava, its pathophysiology has now catapulted into the realm of genetics, which puts into disrepute older hypotheses about nutritional deficiencies and toxic effects of cassava.

**Table 2.** Frequencies of mutations/polymorphisms in major genes in patients with idiopathic Chronic Pancreatitis in India

	*SPINK* gene		*CFTR* gene		*CTRC* gene		*MORC4/CLDN2 locus*	
	Patients	Controls	Patients	Controls	Patients	Controls	Patients	Controls
Chandak *et al*. [32]	39/120 (32.5%)	8/290 (2.76%)						
Bhatia *et al*.[33]	29/66 (44%)	2/90 (2.2%)						
Midha *et al*. [15]	48/113 (42.4%)	4/100 (4%)	9/100 (9%)	0				
Derikx *et al.* [38]	47/150 (31.8%)	7/146 (4.7%)			5.4%	0.7%		
Singh *et al*. [34]	61/150 (41.2%)	3/150 (2%)	49/150 (32.6%)	15/150 (10%)				
Paliwal *et al*. [39]					71/584 (12.2%)	22/598 (3.7%)		
Sundaresan *et al*. [37]	67/174 (38.5%)	10/234 (4.3%)						
Giri *et al*. [40]							OR 1.62	
rs6622126 (*MORC4*)							OR 1.57	
rs4409525 (*CLDN2*)							(Number of patients = 434 Number of controls = 1288)	

Numerator denotes the number of participants tested positive for the mutation/polymorphism out of the total number of participants tested (denominator).

• F508delta mutation.

• OR, odds ratio.

### Environmental influences in the aetiopathogenesis of CP

In complex diseases with gene-environment interactions, it is important to understand the role of environmental factors. One of the mechanisms of chronic inflammation in patients with CP is oxidative stress (OS) [41]. Xenobiotics are detoxified in the body through phase I and phase II pathways chiefly in the liver. Increased exposure to xenobiotics such as alcohol, nicotine, petrochemical fumes may overwhelm the capacity of these detoxification pathways and result in OS in pancreatic acinar cells [42, 43]. OS may lead to inflammation by cell damage and chemotaxis [44]. Increased OS has been demonstrated in patients with alcoholic and idiopathic chronic pancreatitis [45]. Supplementation with antioxidants may result in decreased OS and pain relief, as shown by us previously [46]. Nutrition while not directly leading to the development of CP, may play a role if there is deficiency of micronutrients leading to inefficient antioxidant capacity and increased OS.

Alcohol consumption even in moderate amount and smoking are also known to be associated with CP. In large epidemiological studies, smoking has been found to be an independent risk factor for the development of CP [47]. Studies have shown an increase in the prevalence of smoking and alcohol consumption among patients with CP from Kerala [27].

## How to explain the changing phenotype of CP in India? The impact of environmental influences

The reasons for a change in the phenotype of CP could either be a change in the genetic make-up of individuals or a change in environmental influences. It is highly unlikely that genetic changes would occur over a relatively short period of time i.e. over a few decades. It would take centuries for the genetic make-up of a population to change due to population admixture or *de novo* polymorphisms. On the other hand, it is quite likely that environmental influences may change over such a period of time. Important environmental factors are likely to play a significant role in idiopathic CP since genetic mutations, though necessary, are probably not sufficient to cause CP except probably in the case of hereditary pancreatitis with autosomal dominant inheritance. It is rather unlikely that climatic condition is one such important factor e.g. in Brazil, a tropical country, the profile of chronic pancreatitis is somewhat similar to western population [48].

Nutritional factors seem to be important modulator of the disease phenotype. In this context, protein calorie malnutrition is not related to pathophysiology as discussed above but could be a modifier of the phenotype. Protein calorie malnutrition has been shown to induce pancreatic atrophy. With adequate intake of nutrients (particularly proteins and calories) and normal body mass index (BMI), pancreatic atrophy is not seen now in patients with CP until late stage of the disease unlike in the past.

OS due to increased environmental exposure to xenobiotics and micronutrient deficiencies also play a role in the pathophysiology of CP. With improved nutrition, such OS may be better handled thus resulting in slower disease progression.

Improved sanitation and better nutrition have also led to a decrease in infectious disease morbidity. The reduced co-morbidities burden has also contributed to improved prognosis of patients with CP.

The changes that have had a profound impact on the phenotype of CP can be summarized as follows: higher BMI, less chances of recurrent infections, better antioxidant defence with adequate micronutrient supply, and lesser pancreatic atrophy due to adequate protein and calorie consumption. In addition, greater access to health care and availability of modern diagnostic tests have resulted in diagnosis at an earlier stage of the disease and better treatment has led to improved prognosis.

The above mentioned external influences have been largely driven by rapid socio-economic development of the society in India. In this regard, it is imperative and informative to discuss the socio-economic changes in Kerala over the past 3 decades.

## Kerala: model of socio-economic development

The Kerala Development report prepared by Planning Commission, Government of Kerala has shown that the state gross domestic product (GDP) of Kerala has steadily grown over the past 4 decades. GDP is a measure of the economic health of a region/state/country.

### Income of the state of Kerala

The Net State Domestic Product (NSDP) of Kerala has increased continuously over years with a significant turnaround in the trend around 1987–1988 (Fig. 3) [49]. The net state GDP was Indian Rupees (Rs.) 1457 × 10^7^ (~$320 million) and the *per capita* GDP was Rs. 665 (~$15) in 1972–1973; these figures increased to Rs. 62 557 × 10^7^ (~$13.75 billion) and Rs. 23 865 ($525), respectively in 1999–2000, i.e. an almost 40-fold increase [47]. In addition to the NSDP, foreign remittances received by Kerala households has grown considerably e.g. Rs. 35 304 million ($526 million) in cash and Rs. 5413 million ($81 million) in kind with the total estimated remittances being Rs. 40 717 million ($607 million) bearing a ratio of 10.7% to SDP in 1998. This has increased to 25% by 2010. In general, gross SDP is a poor indicator of consumption by people as it does not include remittances or other factor payments. A better measure would be the proportion of population below the poverty line. With increasing income levels, there has been a decline in the poverty in Kerala. As a measure of overall poverty in the population, the headcount index of poverty has steadily fallen from 59.19 in 1973–1974 to 9.40 in 1999–2000 in Kerala.

### Benefits of increasing NSDP at the population level in Kerala

The life expectancy at birth increased from 44.3 years in 1956 to 70.4 years in 1995 for males and from 45.3 years to 75.9 years for females in Kerala. The human development index of Kerala has increased from 0.685 in 1991 to 0.773 in 2001 [48].

### Relationship between poverty and malnutrition

**Fig. 4.**
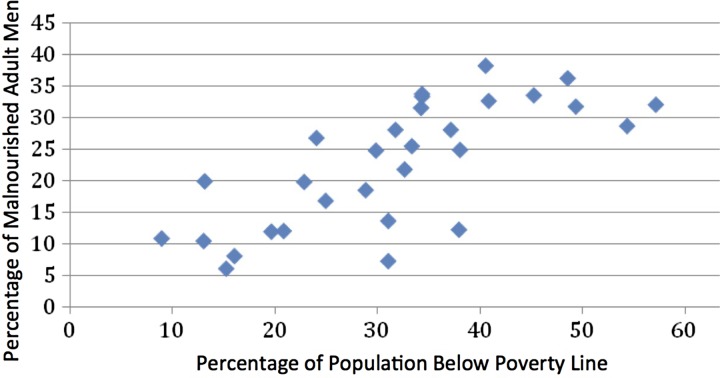
Relationship between poverty and malnutrition in Indian men (source: Rangarajan Committee Report on Poverty and National family Health Survey-3; reference 49).

There is a close relationship between the proportion of population below poverty line and proportion of adults malnourished, as shown in Fig. 4 (correlation coefficient = 0.7314, *P* = 0.0026) (we have taken the proportion of poor for the states in the year 2004–2005 (http://planningcommission.nic.in/reports/genrep/pov_rep0707.pdf); malnourishment is based on below normal BMI).

### Other reasons for improvement in nutrition, human development and better health

In addition to increasing *per capita* income, successive governments in Kerala have introduced a large number of welfare schemes for the people – about 35 social security schemes such as public distribution system, free noon meal at school etc [48]. With increasing net SDP and falling fertility rates, (fertility rates have fallen from 3.7 in the 1970s to 1.7 in 2001 in Kerala), the *per capita* expenditure on various schemes has translated into much more gains in terms of people's welfare.

### Is it hypothetical or is there a real relationship between disease phenotype and socio-economic development?

**Fig. 5.**
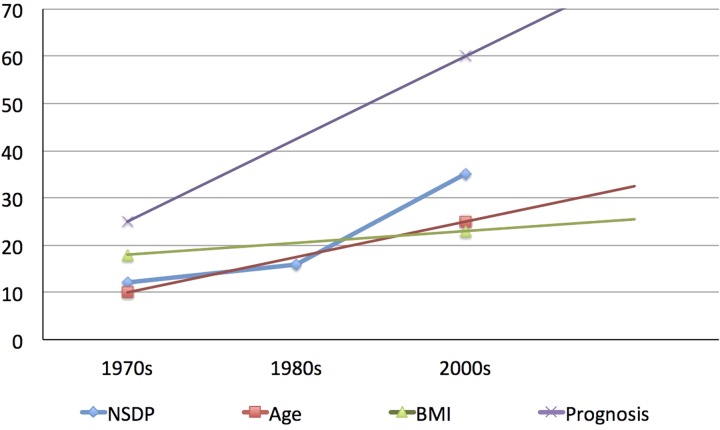
Trends in age at onset, body mass index (BMI), and prognosis (as represented by life expectancy in years) of patients with chronic pancreatitis in relation to the trend in the Net State Domestic Product (NSDP in 10 X billion Indian Rupees) (Modified from Human Development Report: Kerala). The trends in patients’ characteristics have been derived from reference 14 and 15. Although shown as linear, trend may not be linear over years.

The arguments that have been made above regarding the change in phenotype and socio-economic development could just be hypothetical. However, when we look at the data, the relationship acquires a real dimension. Figure 5 shows how the age of onset of CP, the BMI, and the prognosis of CP have evolved in relation to the improving NSDP of Kerala. It can be surmised that the age of onset of disease, BMI of patients, and life expectancy of patients with CP (extrapolated from a study from northern India [15]) have been increasing in parallel to the increasing net SDP. Although the comparison may be prone to bias given the different time periods of different studies and relatively few data points, it is compelling to generate a hypothesis and understand the relationship.

## Negative influence of rising incomes: alcohol consumption as an indicator and its impact on CP

In general, 15–20% of Indian people consume alcohol and the number of drinkers has increased from one in 300 to one in 20 over the past 20 years [49]. The national average *per capita* consumption of alcohol is 4 litres but that of Kerala stands first at 8.3 litres. The age at which people begin to consume alcohol has come down from 19 years in 1986 to 14 years in 1990 in Kerala [49]. This has led to an increase in many social problems: increase in alcohol related diseases, traffic accidents, violence, and mortality. In the context of CP, alcoholic CP has been increasing over time e.g. it accounted for only 2% of all CP in Kerala in the year 1984, which increased dramatically to 33% in 2004 [28].

### Implications of changing phenotype and disease behaviour of CP:

1. Implication for terminology of chronic pancreatitis in India: Is the term ‘tropical pancreatitis’ a misnomer?

Although it is just a matter of semantics, a correct terminology helps convey the right message about disease pathophysiology and/or epidemiology. The word ‘tropical’ usually refers to diseases that are of infectious aetiology. As per WHO, ‘Tropical diseases encompass all diseases that occur solely, or principally, in the tropics. In practice, the term is often taken to refer to infectious diseases that thrive in hot, humid conditions, such as malaria, leishmaniasis, schistosomiasis, onchocerciasis, lymphatic filariasis, Chagas disease, African trypanosomiasis, and dengue’ (http://www.who.int/topics/tropical_diseases/en/). On the other hand, data shown above have established that genetic mutations play an important role in the pathogenesis of idiopathic chronic pancreatitis in India similar to western patients. Thus, the term ‘tropical pancreatitis’ does not conform to the present day understanding of its aetiopathogenesis. Furthermore, it cannot be said that there are 2 different diseases i.e. tropical and idiopathic chronic pancreatitis, both having similar genetic mutational profiles and other characteristics within the same country. If only 3–5% of our patients with idiopathic CP are young, malnourished and diabetic, that does not mean that they constitute a different disease, just a faster progression to an advanced form of CP. Furthermore, there is no perceivable influence of climatic conditions on the pathophysiology of CP. Earlier, about 80–90% of chronic pancreatitis was considered to be alcohol related in western countries but the recent data have shown that only about 38% of chronic pancreatitis might be ascribed to heavy alcohol consumption implying a misclassification bias in previous studies [50]. That means that at least 50–60% of patients have non-alcoholic chronic pancreatitis in western countries having a temperate climate. The question is, ‘should there be a separate term for such an entity e.g. ‘temperate’ chronic pancreatitis’? The term ‘tropical’ makes the disease esoteric, confines it to a geographical location and makes the data non- generalizable. It has been suggested that the term ‘Tropical Pancreatitis’ is a misnomer. Balakrishnan, a gastroenterologist based in Kerala who has worked for long on chronic pancreatitis, noted, ‘As the aetiology was only a matter of speculation and since there were no other diagnostic markers for this disease, for the sake of differentiating it from the well characterized alcoholic pancreatitis, a convenient but inaccurate term of ‘tropical pancreatitis’ was applied to refer to this disease. This term was obviously illogical, as we were comparing alcoholic pancreatitis named so based on aetiology, to tropical pancreatitis, named so on the basis of geographical prevalence’ [51].

Therefore, we suggest that the term ‘tropical pancreatitis’ should be replaced. In view of the strong association between ‘idiopathic CP’ and genetic mutations/polymorphisms in multiple genes such as *SPINK1, CFTR, CTRC, Cathepsin B* and *CLDN2/MORC4* as reported in patients from different countries including India, a newer and more inclusive term such as Chronic Pancreatitis-G where ‘G’ denotes ‘Genetic’ should be considered [52].

1. Implications for treatment: With the knowledge that not all patients with CP have an advanced disease, it is possible that CP may be diagnosed at a much earlier stage through increased awareness and screening of high-risk population. Genetic mutational screening in first-degree relatives might be offered. An earlier diagnosis might help better treatment e.g. by advising against exposure to pollutants and toxins such as alcohol and smoking, replenish their antioxidant capacity by nutritious diet and antioxidant supplementation, and recognition and treatment of complications. However, genetic testing has not been recommended internationally due to concerns such as cost, social stigma, and issues with medical insurance.
2. Implications for decreasing morbidity: With the knowledge that the phenotype of CP has changed due to evolving environmental factors, it offers an explanation to better understand gene–environment interaction in complex diseases. Genetic predisposition is a major risk factor for the large majority of patients with idiopathic CP but the disease behaviour is significantly influenced by environmental factors, in this particular instance by rapid socio-economic development. Population level interventions might be effective for improving outcomes of diseases that are genetically determined or predisposed yet modifiable by environmental factors such as diet, smoking and nutrients.

### Prospects of future research

The areas that future research should focus on include unravelling the functional mechanism(s) of genetic mutations predisposing to development of CP, interaction between genetic polymorphisms and environmental influences in the pathogenesis of CP, and how modifiable environmental factors could alter the natural course of CP.
